# Narrative review of focal boost to intraprostatic dominant lesion in intensity-modulated radiation therapy for localized or locally advanced prostate cancer

**DOI:** 10.1007/s10147-025-02799-x

**Published:** 2025-06-08

**Authors:** Rihito Aizawa, Takashi Mizowaki

**Affiliations:** Department of Radiation Oncology and Image-Applied Therapy, Graduate School of Medicine, Kyoto University, 54 Shogoin Kawahara-cho, Sakyo-ku, Kyoto, 606-8507 Japan

**Keywords:** Prostate cancer, Intensity-modulated radiation therapy, Focal boost, Intraprostatic dominant lesions

## Abstract

Local intraprostatic recurrence is one of the important recurrence patterns following definitive intensity-modulated radiation therapy (IMRT) that continues to pose a significant challenge. The technological advances in radiation therapy now facilitate selective dose escalation for intraprostatic dominant lesions (IPDLs). A novel IMRT method, focal boosting using simultaneous integrated boost IMRT (FB-SIB-IMRT), can achieve selective dose escalation to IPDLs while minimizing any increase in dose to organs at risk (OARs). In addition, this method is applied to hypofractionated EBRT, including stereotactic body radiation therapy (SBRT). To date, numerous prospective studies have reported clinical results of FB-SIB-IMRT for non-metastatic PCa. In this review, we describe and summarize clinical outcomes of previous studies, including the technological background, current status, and future perspectives regarding focal dose escalation for IPDLs using IMRT for non-metastatic PCa.

## Introduction

Prostate cancer (PCa) is globally the second most common malignant tumor in males [1], and the incidence rate continues to increase [2]. Definitive external-beam radiation therapy (EBRT) is one of the established treatment modalities for localized or locally advanced PCa [3, 4]. The marked technological advances in EBRT of this century include intensity-modulated radiation therapy (IMRT) and image-guided radiation therapy (IGRT). IMRT is an evolved and developed form of standard EBRT, which enables the radiation dose to be increased safely by selectively protecting a significant volume of organs at risk (OARs) from high-dose radiation [5]. In addition, IGRT, an essential technique to correct geographic errors in target-positioning during the treatment course, markedly improves the accuracy of dose delivery to the target. The combination of these two technological innovations, IMRT and IGRT, has further expanded the role of EBRT in curative treatment for patients with PCa.

Local intraprostatic recurrence remains an important recurrence pattern following definitive EBRT that needs to be resolved. Although dose-escalation to improve local control is required, simply elevating the dose would increase RT-related toxicities, such as rectal bleeding, dysuria, and hematuria. The technological advances in EBRT, particularly IMRT and IGRT, enable selective dose escalation for intraprostatic dominant lesions (IPDLs). This novel IMRT method, focal boosting using simultaneous integrated boost IMRT (FB-SIB-IMRT), can achieve selective dose escalation to IPDLs while minimizing the dose increase to OARs. The FLAME phase 3 trial demonstrated that this novel IMRT method improved tumor control without increasing toxicities [6]. In addition, hypofractionation, including stereotactic body radiation therapy (SBRT), was applied to FB-SIB-IMRT. To date, numerous prospective studies have published promising clinical results of FB-SIB-IMRT for non-metastatic PCa. However, despite this, its introduction into daily clinical practice has been limited [7].

Here, we reviewed the clinical outcomes of previous studies, including the technological background, current status, and future perspectives regarding focal dose-escalation for IPDLs using IMRT for patients with non-metastatic PCa.

## Rationale behind efficacy of focal dose escalation in IMRT

Local intraprostatic recurrence remains an important recurrence pattern following definitive EBRT. Regarding the location of intraprostatic recurrence, several retrospective studies investigated the spatial pattern association between primary and recurrent tumor sites [8–11]. Most studies reported that recurrent tumors originated at the same site as primary IPDLs based on pathological findings or magnetic resonance imaging (MRI) investigations. Arrayeh et al. investigated the spatial pattern association among nine patients who developed local recurrence following definitive EBRT (74–79 Gy) using MRI and magnetic resonance spectroscopic imaging, and reported that the dominant recurrent tumor was located at the same site as the primary IPDL in 89% [8]. More recently, Raman et al. analyzed recurrence patterns after brachytherapy with or without EBRT using prostate-specific membrane antigen-targeted (PSMA) positron emission tomography/computed tomography (PET/CT) [10]. Among 42 patients who developed intraprostatic recurrence, recurrent tumors were found within the same sextant of the prostate as pathologically included in the initial diagnostic biopsy in 69.0%.

We retrospectively analyzed the spatial pattern association between primary and recurrent tumor sites among 12 patients who developed local recurrence following definitive EBRT [11]. We independently re-evaluated the location of the primary IPDLs on MRI at the initial diagnosis and intraprostatic site of recurrence detected by PSMA-PET/CT [12]. As a result, the recurrent tumor was found at the same location or a partially overlapping site adjacent to the primary IPDLs in 66.7%.

Those observations formed the basis behind the rationale of focal dose-escalation for IPDLs in definitive EBRT.

## Overview of prospective trials

In the early 2010 s, data from prospective studies that attempted focal dose-escalation using SIB-IMRT technique were published, indicating that FB-SIB-IMRT is a promising method to achieve safe dose-escalation to IPDLs [13–15]. Ippolito et al. conducted a phase 1–2 study to assess the feasibility of FB-SIB-IMRT among 40 patients with T2–3 N0M0 PCa. The prescribed dose was 72 Gy (1.8 Gy per fraction) to the prostate and seminal vesicles, increased to 80 Gy for MRI-visible IPDLs [13]. Grade 3 acute gastrointestinal (GI) and genitourinary (GU) toxicities were observed only in 5 and 2.5%, respectively, and no grade 4 acute toxicities were noted. The 2-year cumulative incidences of ≥ grade 2 rectal and urinary toxicities were 9.5 and 13.3%, respectively. Similarly, in 2011, Wong et al. reported the 5-year results of a prospective feasibility study of FB-SIB-IMRT, in which 75.6 and 82 Gy in 42 fractions were simultaneously prescribed to the entire prostate and IPDLs, respectively. Although the incidence of late GI and GU toxicities was relatively high (21% for grade 2 GI toxicities; 39% for grade 2, 4% for grade 3, and 1.4% for grade 4 GU toxicities), most AEs improved with a longer follow-up duration [14].

In 2020, the landmark FLAME phase 3 trial established an essential benchmark of superior biochemical control using FB-SIB-IMRT compared with conventional IMRT [6]. A total of 571 patients with intermediate- to high-risk PCa received 77 Gy in 35 fractions (2.2 Gy per fraction) to the entire prostate with or without focal dose-escalation up to 95 Gy (2.7 Gy per fraction). This trial demonstrated significant improvement in biochemical disease-free survival without increasing toxicities (*p* < 0.001).

Owing to the low alpha–beta ratio of PCa (in the range of 1–2 Gy) compared with normal tissues [16], the application of hypofractionated EBRT indicates the merit of improving the therapeutic ratio, enabling the delivery of a higher biological equivalent dose (BED) to the prostate without increasing that to adjacent normal tissues. In addition, the hypofractionated EBRT also has merits regarding patient convenience and medical economics. Such merits have accelerated the development and subsequent clinical application of hypofractionation in prostate EBRT. According to the hypofractionated EBRT guidelines for localized PCa published by the American Society for Radiation Oncology (ASTRO), the American Society of Clinical Oncology (ASCO), and the American Urological Association (AUA), the fractionation size is classified into conventional fractionation (1.8–2 Gy per fraction), moderate hypofractionation (2.4–3.4 Gy per fraction), and ultra-hypofractionation (≥ 5 Gy per fraction) which is mainly performed using the SBRT technique [17]. In the mid-2010 s, the results of several phase 3 trials comparing hypofractionated EBRT with conventional fractionated EBRT for non-metastatic PCa were published, in which the non-inferiority of moderate hypofractionated RT was demonstrated [17]. In addition, the results of two phase 3 trials evaluating the efficacy of SBRT were published in 2019 and 2024 [18, 19]. The phase 3 HYPO-RT-PC trial compared SBRT (42.7 Gy in seven fractions, 3 days per week) with conventional fractionated RT mainly for IR-PC, and demonstrated the noninferiority of SBRT in terms of failure-free survival without increasing RT-related toxicities, except for a tendency to increase acute grade 2 or higher GU toxicities [18]. The other phase 3 PACE-B trial, comparing SBRT (36.25 Gy in 5 fractions over 1–2 weeks) with conventional fractionated or moderately hypo-fractionated RT (78 Gy in 39 fractions or 62 Gy in 20 fractions) mainly for intermediate-risk PCa, also reported noninferiority with respect to tumor control [19]. In Japan, hypo-fractionated EBRT has been widely applied in daily clinical practice, especially since the COVID-19 pandemic. Of note, national insurance system approval of SBRT application for localized PCa in 2016 promoted the expansion of its use, and several institutions reported their initial or medium-term experience [20–25]. This trend of hypofractionation in prostate EBRT has also been applied in the development of FB-SIB-IMRT.

Regarding FB-SIB-IMRT using moderate hypofractionation, the medium-term results of prospective studies were published from the late 2010 s to 2020. Onjukka et al. conducted a prospective study to assess the feasibility of moderately hypofractionated FB-SIB-IMRT. The prescribed dose was 60 Gy to the prostate and increased to 68 Gy to IPDLs in 20 fractions, and this dose fractionation schedule demonstrated an acceptable safety profile [26]. The DELINEATE trial was a phase 2 multicohort study evaluating the role of focal dose-escalation among intermediate- or high-risk PCa using conventional or moderately hypofractionated IMRT [27]. Their moderate hypofractionation cohort received a similar dose fractionation. A total of 153 patients were included in the moderate hypofractionated IMRT and received FB-SIB-IMRT with a dose of 60 Gy to the entire prostate and 67 Gy to IPDLs in 20 fractions. According to their long-term follow-up data published in 2023, rates of severe EBRT-related toxicities were low throughout the follow-up [28]. Specifically, 5-year cumulative rates of grade 3 GI and GU late toxicities were limited to 0.9 and 2%, respectively, and no grade 4 or higher toxicities were observed.

Regarding FB-SIB-IMRT using ultra-hypofractionation, the results of prospective trials, mainly describing early-phase toxicity data, were published from around 2020, in which SBRT was applied as an EBRT technique [29–39]. The phase 2 hypo-FLAME trial was the key prospective trial of SBRT with focal dose-escalation to IPDLs, evaluating the safety of this treatment method [29]. One-hundred patients with intermediate- to high-risk PCa received 35 Gy in 5 once-weekly fractions to the entire prostate with an integrated boost up to 50 Gy to IPDLs. Later, they performed another phase 2 hypo-FLAME 2.0 trial to investigate the safety of an alternative EBRT schedule with the same prescribed dose [39]. More specifically, a semi-weekly schedule was used, shortening the overall treatment time from 29 to 15 days. Another phase 2 SPARC trial also evaluated the safety of SBRT with focal-dose escalation among unfavorable intermediate- and high-risk PCa patients, in which a similar dose and fractionation schedule, 36.25 Gy in 5 fractions (alternate days) to the entire prostate with an integrated boost of 47.5 Gy to IPDLs, was used [32, 33]. At present, the HypoFocal-SBRT phase 3 trial (DRKS00022915) is ongoing to assess the benefit of FB-SIB-IMRT with SBRT over moderately hypofractionated IMRT without a focal boost [40].

**Fig. 1:**
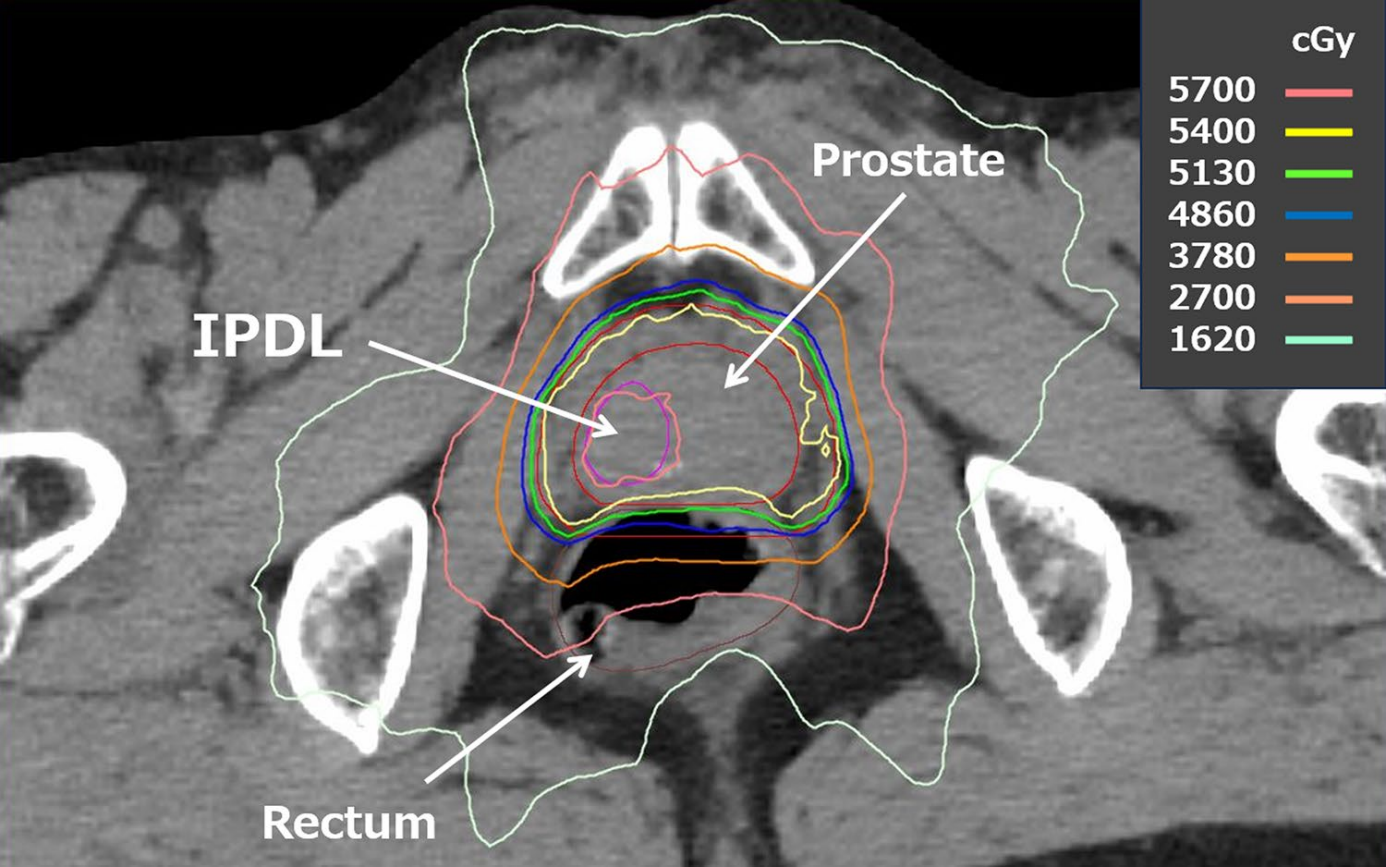
An example of structure delineation and dose distribution of focal boosting using simultaneous integrated boost intensity-modulated radiation therapy. A dose of 54 Gy in 15 fractions was prescribed for the prostate in combination with focal boost for intraprostatic dominant lesions at a dose of 57 Gy in 15 fractions. *IPDLs* intraprostatic dominant lesions

The fraction size between moderate hypofractionation and ultra-hypofractionation (3.4–5 Gy per fraction) has rarely been investigated. Therefore, there is a gap in evidence and knowledge between the two dose fractionations in the aforementioned hypofractionated radiation therapy guidelines. Our institution previously conducted a prospective pilot study of hypofractionated IMRT for patients with low- and intermediate-risk PCa using dose fractionation between those two categories (3.6 Gy per fraction), termed “high hypofractionation,” in which 54 Gy in 15 fractions was prescribed over 3 weeks, and reported its usefulness and safety data [41, 42]. Based on the data, we performed a prospective pilot study to evaluate the feasibility of FB-SIB-IMRT for high-risk patients according to the D’Amico classification [43] or ≥ T3a cases using this “high hypofractionation,” in which a dose of 54 Gy in 15 fractions was prescribed for the prostate in combination with SIB for IPDLs at a dose of 57 Gy in 15 fractions [44] (Fig. 1). To our knowledge, no other prospective study has reported the clinical outcomes of hypofractionated EBRT, mainly for patients with high- and very high-risk PCa, with focal boosting for IPDLs employing a fraction size between moderate and ultra-hypofractionation.

The summary of prospective studies of FB-SIB-IMRT are shown in Tables 1 and 2.

**Table 1:** Summary of the results of prospective studies of FB-SIB-IMRT using conventional fractionation and moderate or high hypofractionation

References	Reference number	Study design	Risk of population	*N*	Type of fractionation	IPDL deliniation	Marigins for IPDLs	Dose for the IPDLs (Gy/fr)	Dose for the prostate gland (Gy/fr)	Median *f*/*u*	5-year bDFS	Acute toxicities (≥ G2)	Acute toxicities (≥ G3)	Late toxicities (≥ G2)	Late toxicities (≥ G3)	Comment
Kerkmeijer et al. FLAME study (2021)	[6, 84]	Phase 3	IR–HR	Arm 1: 284 Arm 2: 287	CF	MRI	0 mm	Arm 1: 95 Gy/35 fr Arm 2: no boost	77 Gy/35 fr	72 months	92% 85% *p* < 0.001	GU: 42.3%, GI: 14.8% GU: 46.0%, GI: 10.1%	N/A N/A	GU: 27.8%, GI: 12.7% GU: 23.0%, GI: 12.2%	GU: 5.6%, GI: 1.4% GU: 3.5%, GI: 1.4%	
Tree et al. DELINEATE study (2023)	[27, 28]	Phase 2	IR–HR	Cohort A: 55 Cohort B: 153 Cohort C: 48	CF MHF CF	MRI	2 mm	Cohort A: 82 Gy/37 fr Cohort B: 67 Gy/20 fr Cohort C: 82 Gy/37 fr	74 Gy/37 fr 60 Gy/20 fr 74 Gy/37 fr	> 60 months	98.2% 96.7% 95.1%	GU: 38.2%, GI: 10.9% GU: 35.9%, GI: 13.7% GU: 37.5%, GI: 14.6%	≤ 2% One patient in the cohort B developed G4 GU toxicity (urinary catheter)	GU: 12.9%, GI: 12.8% GU: 18.2%, GI: 14.6% GU: 18.2%, GI: 20.7% (5-y cumulative incidence of G2 toxicities)	GU: 3.7%, GI: 0% GU: 2%, GI: 0.9% GU: 2.5%, GI: 2.7% (5-y cumulative incidence of G3 toxicities)	No more than 3 separate IPDLs were permitted to be boosted WPRT was used in the cohort C
Zapatero et al. (2022)	[74]	Phase 2	IR–HR	30	CF	MRI	2–3 mm	85.05 Gy/35 fr	75.95 Gy/35 fr	30 months	N/A	GU: 20%, GI: 0%	GU: 0%, GI: 0%	GU: 0%, GI: 0% (occurrence rate during f/u)	GU: 0%, GI: 0% (occurrence rate during f/u)	
Buwenge et al. (2020,2012)	[13, 59]	Phase 1/2	IR–HR	44	CF	MRI	15 mm	80 Gy/40 fr	72 Gy/40 fr	120 months	95.3%	GU: 30%, GI: 15%	GU: 2.5%, GI: 5%	GU: 90.9%, GI: 86.2% (5-y rates for freedom from toxicities)	GU: 95.5%, GI: 97.7% (5-y rates for freedom from toxicities) one patient developed G4 GU toxicity	IPDLs involving < 75% of the prostate gland
Schild et al. (2017, 2011)	[14, 82]	Prospective	All risks	71	CF	ProstaScint	0 mm	Arm 1: 82 Gy/35 fr Histrical control: no boost	75.6 Gy/42 fr	120 months	94% (5-y), 85% (10-y) 86% (5-y), 61% (10-y) *p* = 0.02	GU: 56%, GI: 49%	GU: 0%, GI: 0%	GU: 44%, GI: 15%	GU: 5%, GI: 0% one patient developed G4 GU toxicity	
Dankulchai et al. (2022)	[73]	Prospective	IR–HR	45	CF MHF	MRI	3–5 mm	87.75 Gy/39 fr 70 Gy/20 fr	78 Gy/39 fr 60 Gy/20 fr	20 months	N/A	GU: 33.1%, GI: 9.5%	GU: 2.2%, GI: 2.2%	GU: 12.6%, GI: 2.8%	GU: 0%, GI: 0%	
Onjukka et al. (2017)	[26]	Prospective	HR	28	MHF	MRI	5 mm	68 Gy/20 fr	60 Gy/20fr	37 months	N/A	N/A	N/A	GU: 7.1%, GI: 0%	GU: 0%, GI: 0%	
Camden et al. (2024)	[45]	Phase 2	all risks	50	CF and UHF (boost)	MRI	1–2 mm	45 Gy/25 fr plus 18 Gy/3 fr	21 Gy/3 fr	43.5 months	N/A	GU: 40%, GI: 10%	GU: 0%, GI: 0%	GU: 53%, GI: 4% (Grade 2 at 18 months)	GU: 2%, GI: 0% (at 18 months)	SBRT focal boost following CF RT for prostate gland
Uzan et al. (2016)	[15]	Prospective	HR	11	CF	MRI	5 mm	75.4 Gy/37 fr	86 Gy/37 fr	36 months	N/A	GU: 36.4%, GI: 0%	GU: 0%, GI: 0%	GU: 18.2%, GI: 9.1%	GU: 0%, GI: 0%	
Aizawa et al. (2025)	[44]	Prospective	IR–HR	26	High hypofractionation	MRI	3 mm	57 Gy/15fr	54 Gy 15fr	49.7 months	75.2%	GU: 26.9%, GI: 7.7%	GU: 0%, GI: 0%	GU: 15.4%, GI: 3.8% (at 5-y)	GU: 0%, GI: 0%	

*FB-SIB-IMRT* focal boosting using simultaneous integrated boost intensity-modulated radiation therapy, *IPDLs* intraprostatic dominant lesions, bDFS biochemical disease-free survival, *LR* low-risk, IR intermediate-risk, *HR* high-risk, *CF* conventional fractionation, *MHF* moderate hypofractionation, *UHF* ultra-hypofractionation, *MRI* magnetic resonance imaging, *GU* genitourinary, *GI* gastrointestinal *N/A* not available, *SBRT* stereotactic body radiation therapy

**Table 2:** Summary of the results of prospective studies of FB-SIB-IMRT using ultra-hypofractionation

Reference	Reference number	Study design	Risk of population	N	Type of fractionation	IPDL delineation	Margins for IPDLs	Dose for the IPDLs (Gy/fr)	Dose for the prostate gland (Gy/fr)	Median *f*/*u*	bDFS	Acute toxicities (≥ G2)	Acute toxicities (≥ G3)	Late toxicities (≥ G2)	Late toxicities (≥ G3)	Comment
Draulans et al. Hypo-FLAME study (2020, 2024)	[29, 61]	Phase 2	IR–HR	100	UHF	MRI	0 mm	Up to 50 Gy/5 fr (weekly)	35 Gy/5 fr (weekly)	61 months	93% at 5-y	GU: 34%, GI: 5%	GU: 0%, GI: 0%	GU: 28%, GI: 14%	GU: 2%, GI: 1%	
Herrera et al. (2019, 2023)	[30, 37]	Phase 1a/b	IR–HR	20	UHF	MRI	3 mm	40–50 Gy/5 fr (no more than 2 fr per week)	36.25 Gy/5fr (no more than 2 fr per week)	61 months	69% (biochemical control rate)	GU: 25%, GI: 5%	GU: 0%, GI: 0%	GU: 12.1%, GI: 3%	GU: 0%, GI: 0%	
Marvaso et al. AIRC IG-13218 study (2020)	[31]	Phase 2	LR–IR	65	UHF	MRI	3 mm	37.5 Gy/5fr (every other day)	36.25 Gy/5fr (every other day)	24 months	97% at 2-y	GU: 6.2%, GI: 0% (G2 toxicities at 1 months)	GU: 1.5%, GI: 0% (G3 toxicities at 1 months)	GU: 4.7%, GI: 1.7% (G2 toxicities over 6 months)	GU: 0%, GI: 1.7% (G3 toxicities over 6 months)	
Yasar et al. SPARC study (2024)	[33]	Phase 2	IR–HR	20	UHF	MRI	0 mm	Up to 47.5 Gy/5fr (on alternative days)	36.25 Gy/5fr (on alternative days)	30 months	N/A	GU: 25%, GI: 30%	GU: 5%, GI: 0%	GU: 16.7%, GI: 0%	GU: 0%, GI: 0%	Late toxicities were evaluated among 12 patients who were included in late toxicity analysis
Deodato et al. DESTROY-4 study (2024)	[34]	Phase 1	LR–IR	24	UHF	MRI	3 mm	40–50 Gy/5 fr	35 Gy/5 fr	26.3 months	N/A	GU: 12.5%, GI: 12.5%	GU: 0%, GI: 0%	GU: 4.2%, GI: 12.5%	GU: 0%, GI: 0%	
Maas et al. (2023)	[36]	Prospective	LR–IR	26	UHF	MRI	3–5 mm	40 Gy/5fr (during 7–17 days)	36.25 Gy/5fr (during 7–17 days)	59.5 months	100% (at end of f/u)	7.7% needed temporary catheter	GU: 0%, GI: 0%	GU: 38.5%, GI: 11.5%	GU: 0%, GI: 0%	
Ong et al. 2SMART study (2023)	[38]	Phase 2	LR–IR	30	UHF	MRI	0 mm	32 Gy/2 fr (weekly)	26 Gy/2 fr (weekly)	44 months	96.7% (at end of f/u)	GU: 56.7%, GI: 3.3%	GU: 0%, GI: 0%	GU: 50%, GI: 10%	GU: 0%, GI: 0%	
Cock et al. Hypo-FLAME 2.0 study (2023)	[39]	Phase 2	IR–HR	124	UHF	MRI	0 mm	up to 50 Gy/5 fr (sei-weekly)	35 Gy/5 fr (sei-weekly)	N/A	N/A	GU: 47.5%, GI: 7.4%	GU: 0%, GI: 0%	N/A	N/A	

*FB-SIB-IMRT* focal boosting using simultaneous integrated boost intensity-modulated radiation therapy, *IPDLs* intraprostatic dominant lesions, *bDFS *biochemical disease-free survival, *LR* low-risk, *IR* intermediate-risk, *HR* high-risk, *UHF* ultra-hypofractionation, *MRI* magnetic resonance imaging, *GU *genitourinary, *GI* gastrointestinal, *N/A* not available

## IMRT methods

Most previous studies used the SIB-IMRT method for focal dose-escalation. In some of them, it was performed via sequential boosting using SBRT following initial EBRT for the entire prostate [45]. As an IMRT method, although we previously used conventional static-port IMRT, volumetric-modulated arc therapy (VMAT), a combined and developed form of IMRT and rotation irradiation technique, is dominant at present. Compared with conventional static IMRT, it was suggested that VMAT has the potential to improve dose distribution in FB-SIB-IMRT compared with conventional static IMRT [46]. Conventional and moderately hypofractionated EBRT were mainly performed using conventional C-arm linac. Ultra-hypofractionated EBRT or SBRT has been mainly performed using CyberKnife® (Accuray Inc., Sunnyvale, California, USA), which is a non-coplanar robotic arm-based EBRT platform primarily focusing on stereotactic radiotherapy. Currently, conventional C-linac based-IMRT, particularly VMAT, is also frequently used. VMAT is more readily available in daily clinical practice because it can be performed via traditional C-linac, and it has the merit of reducing the EBRT-delivery time compared with static IMRT (step-and-shoot IMRT). Considering that intrafractional prostate motion increases with the treatment time [47, 48], VMAT can potentially minimize this motion error compared with static IMRT.

## Delineation of GTV

The gross tumor volume (GTV) was defined as visible IPDLs on multiparametric MRI (mpMRI) according to the prostate imaging-reporting and data system (PI-RADS) in most studies [49], which has been regarded as a gold standard for IPDLs delineation. Recent studies indicated that novel imaging modalities or techniques can improve the accuracy of detection or delineation of IPDLs [50–54]. PSMA-PET is one of the most promising imaging modalities for adding complementary information on mpMRI in IPDLs delineation [55]. According to a comparative study of the diagnostic performance of gallium 68 (^68^Ga) PSMA-PET/MRI for the localization of primary PCa with mpMRI and PET alone, the diagnostic accuracy of PET/MRI statistically outperformed both mpMRI (*p* < 0.001) and PET (*p* = 0.002) [50]. Noteworthily, according to another investigation that compared GTV delineation based on ^68^Ga PSMA-PET with that based on mpMRI, that using PET delineated approximately twice the volume of GTV based on mpMRI (median 4.9 vs. 2.8 mL, respectively, *p* < 0.0001) [52]. Further investigations, such as a comparison of the results from those modalities with histopathology data, are warranted to elucidate which more accurately reflects the actual tumor volume. Therefore, it is considered that using both PSMA-PET and mpMRI may improve delineation of GTV in focal boosting. Early PSMA-PET/MRI-guided focal-boost results have been reported [56, 57]. In addition, as aforementioned, the ongoing HypoFocal-SBRT phase 3 trial has employed PSMA-PET/MRI for the delineation of IPDLs [40]. A quantitative diffusion MRI biomarker, the restriction spectrum imaging restriction score (RSIrs), is another promising technique that has been shown to improve diagnostic utility compared with conventional MRI [58]. According to a multi-institutional, international, prospective study, which tested whether RSIrs maps could improve radiation oncologists’ accuracy of PCa tumor delineation, 91% of participating radiation oncologists completely missed ≥ 1 expert-defined target lesion without RSIrs, while the rate improved to 30% with RSIrs maps [54]. Their results suggested that targeting prostate tumor only with mpMRI can be very difficult for radiation oncologists. Such novel imaging modalities or techniques may provide useful complementary information on mpMRI in the process of IPDLs delineation.

## Target volume and OARs

Target volumes in FB-SIB-IMRT basically consisted of GTV, clinical target volume (CTV), and planning target volume (PTV) for the prostate (with or without seminal vesicles).

As abovementioned, GTV was defined as IPDLs basically detected on mpMRI or using other modalities. In cases with multiple IPDLs or those with a large IPDL volume, dose-escalation to IPDLs may inadvertently increase toxicities because the dose for the whole prostate would become too elevated. In this regard, setting an upper limit for the boost volume would be advisable. Indeed, boost was permitted for no more than 3 separate nodules, although no maximum boost volume was stipulated in the DELINEATE phase 2 trial [27, 28]. Similarly, an Italian phase 2 trial limited the IPDL volume to < 75% of the prostate [13, 59].

CTV for the prostate was defined as the whole gland of the prostate with part of seminal vesicles (dependent on the risks). CTV for IPDLs was not defined or equal to GTV in most studies.

The PTV margin for CTV of the prostate used in conventional or moderate hypofractionation ranged between 5 and 10 mm in conventional or moderately fractionated EBRT, and between 2 and 5 mm in SBRT. The PTV margin is highly dependent on the IGRT method.

The PTV margin for GTV (IPDLs) was not clearly defined. Most PTV margins for GTV were 0–5 mm in conventional or moderate hypofractionation, and 0–3 mm in SBRT. One conventional hypofractionation study used an exceptionally wide margin of 13–15 mm, with the dose to IPDLs (PTV boost) being relatively low (80 Gy in 40 fractions) [13, 59]. Similarly, although one study of SBRT used a 5-mm margin, the dose to IPDLs was relatively low compared with other SBRT studies (40 Gy in 5 fractions) [35, 36]. According to our previous dosimetric analysis evaluating the effect of intrafractional prostate motion on FB-SIB-IMRT, the prostate motion within 30 s mainly reflected respiration-induced movement, and mean displacements of base drift were 1.4, −2.2, and −0.3 mm in the anterior–posterior, superior-inferior, and left–right directions, respectively [60], suggesting that a 3-mm margin around IPDLs is theoretically necessary to compensate for any intrafractional error. However, some studies did not apply a PTV margin around IPDLs; of note, FLAME phase 3, hypo-FLAME phase 2, and hypo-FLAME 2.0 trials did not add such a margin (0 mm for PTV-GTV margin) [6, 29, 39, 61]. Given the known expansion of the high-dose area peripheral to GTV, this approach is considered reasonable in practice from the viewpoint of a high-dose-focus on IPDLs while maintaining dose constraints for OARs, such as the rectum or urethra. Especially, when a higher dose is prescribed to IPDLs, this method may be practical for maintaining the balance between enhancing tumor control and avoiding any increase in toxicities.

Regarding OARs, most studies included the rectum, bladder, and bowels, and some studies which mainly used SBRT defined dose constraints for the urethra or penile bulb [32, 34, 39, 40, 61].

## Method of image guidance

Currently, the use of daily IGRT has become the standard of care in prostate EBRT [5]. Prostate position-based IGRT (P-IGRT) via direct or indirect visualization of the prostate is the current gold-standard method of IGRT in prostate EBRT. P-IGRT methods include indirect visualization of the prostate position using orthogonal radiographs with implanted fiducial makers, and direct visualization of the prostate using cone-beam CT (CBCT), ultrasound, or MRI. As P-IGRT could minimize the day-to-day prostate-positioning error between each treatment session (inter-fractional error) due to bladder and rectal filling, P-IGRT can improve the actual dose delivered to the prostate and reduce dose to normal tissues; therefore, theoretically, it has a positive impact on tumor control [62–64], and can reduce EBRT-related toxicities [63–66].

According to a phase 3 trial by de Crevoisier et al., which compared clinical outcomes of daily vs. weekly P-IGRT among patients with non-metastatic PCa, daily P-IGRT led to a significant improvement in the biochemical failure (BF)-free interval (HR [hazard ratio]: 0.45; 95% CI [confidence interval] 0.25–0.80, *p* = 0.007) [63]. Similarly, according to our previous retrospective study, which evaluated the impact of P-IGRT among patients who received IMRT for intermediate-risk PCa, the BF-free rate was significantly more favorable among patients who received P-IGRT compared with those receiving pelvic bony structure-based IGRT (HR: 3.31; 95% CI 1.10–9.97, *p* = 0.034) [64]. In FB-SIB-IMRT, enhancing dose delivery accuracy is indispensable due to the application of high doses to small volumes. As the P-IGRT modality, IGRT using intraprostatic fiducial markers or CBCT is the most frequently employed. Of note, the intraprostatic fiducial markers have been the most commonly used among studies on SBRT, although their application is invasive and necessitates an implantation procedure. CBCT is also a frequently used IGRT method, being advantageous owing to its marked availability. However, due to the low soft-tissue contrast of CBCT, caution is necessary regarding margin reduction. MRI-guided IGRT is a sophisticated IGRT method with improved soft-tissue contrast. According to the MIRAGE phase 3 trial, which compared MRI- with CT-guided SBRT for PCa, a reduced PTV margin was used in the MRI-guided SBRT arm (2 mm in MRI- vs. 4 mm in CT-guided SBRT arm), and demonstrated significant reduction of acute ≥ grade 2 GU (*p* = 0.01) and GI (*p* = 0.003) toxicities [67]. However, excessive reduction of the PTV margin may result in impaired tumor control. According to a retrospective analysis by Engels et al., which evaluated the impact of PTV margins among patients treated with daily IGRT using implanted fiducial markers, freedom from BF was significantly poorer among patients treated with a smaller PTV margin (3 mm latero-lateral, 5 mm antero-posterior, and 4 mm cranio-caudal) rather than a larger margin (6 mm isotropically) (5-year freedom from BF: 96 vs. 74%, respectively, *p* = 0.04) [68]. Therefore, an adequate PTV margin should be applied to maintain the balance between tumor control and toxicities, in consideration of the method of IGRT, treatment delivery time, and tumor risks.

A peri-rectal hydrogel spacer is used to temporarily create distance between the rectum and prostate, facilitating rectal dose reduction. Based on its dosimetric and clinical benefits, the hydrogel spacer is currently used widely in clinical practice, especially in SBRT cases. However, current IGRT offers excellent safety even without such interventional procedures. According to the H-IGRA phase 2 study of moderately hypofractionated IMRT (70 Gy in 28 fractions) in which a hydrogel spacer was not used in any patients, the incidence of grade 2 GI toxicities was low (5.3% at 5 years), and no ≥ grade 3 toxicities were observed [69]. Similarly, according to retrospective analysis of ultra-hypofractionated FB-SIB-IMRT for 520 patients with localized PCa, in which SBRT (35–36.25 Gy in 5 fractions and doses to IPDLs were elevated up to 115–140% of those prescribed) was performed without hydrogel spacers or fiducial markers, grade 2 late GI toxicities were observed in only 0.8% at 4 years, and no ≥ grade 3 toxicities were noted [70]. Considering the risk of severe toxicities related to hydrogel spacer use [71], further investigations to identify optimal candidates and settings for hydrogel spacer application are of particular importance.

Online adapted radiotherapy (oART) is one of the key technical innovations among IGRT methods, which allows modification of IMRT plans according to inter-fractional (daily) changes in the shape or anatomical geography of the target volumes and OARs. This novel IGRT method has recently been applied to prostate IMRT, which has shown a potential to reduce acute toxicities in prostate SBRT [72].

## Dose prescription

Among studies of conventional or moderately hypofractionated FB-SIB-IMRT, the prescribed dose (calculated with alfa-beta ratio of 1.5 Gy) ranged between the equivalent dose in 2 Gy per fraction (EQD2) of 67.9–81.4 Gy (biological effective dose [BED]: 158.4–190.0 Gy) for the prostate and between EQD2 of 80–114.4 Gy (BED: 186.7–267.0 Gy) for IPDLs (GTV or PTV) in 20–42 fractions, respectively [6, 13–15, 26, 28, 73, 74].

Among studies of ultra-hypofractionated FB-SIB-IMRT (SBRT), most used 5 fractions, in which the prescribed dose to the prostate (PTV) and IPDLs (GTV or PTV) ranged between 35–36.5 and 40–50 Gy, respectively [30, 31, 33, 34, 36, 37, 39, 40, 61]. One phase 2 study used 2 fractions, in which the prescribed dose to the prostate (PTV) and IPDLs (GTV or PTV) was 26 and 32 Gy, respectively [38]. In those SBRT trials, as EBRT was not performed in daily fractions (e.g., once-weekly or semi-weekly fractions), doses used in those trials could not be simply translated into EQD2 or BED.

The optimal dose to IPDLs was not clearly established. According to the meta-analysis by Zaorsky et al., who investigated the association of BED via various radiation fractionation regimens/methods and clinical outcomes, an increase in BED_1.5_ to 200 Gy was associated with improved biochemical control in patients with high-risk PCa [75]. Additionally, according to dosimetric analysis of 24 patients who developed intraprostatic recurrence in the FLAME phase 3 trial, the site of recurrence originated from IPDLs in 96%, and the near minimum dose to GTV (D98% [dose receiving 98% of the volume]) was less than 81 Gy (BED_1.5_ of 205.9 Gy) in all except one case [76]. Given these results, the prescribed dose to IPDLs should be increased to at least above BED_1.5_ of 200–210 Gy. In addition, with an alpha–beta ratio of PCa (reported as in the range of 1–2 Gy) lower than normal tissue (3 Gy) [16], the use of hypofractionation is considered more effective to achieve safe dose-escalation to IPDLs.

## Dose constraints for OARs

Most studies included the rectum, bladder, urethra, small or large bowel, and pineal bulb as OARs. In this review, DX% and DX cc describe the dose to X% and X cc of the target or OAR volume, respectively.

Regarding GU toxicities, in the post-hoc analysis of the FLAME phase 3 trial, the doses (Gy) to the bladder D2 cc and urethra D0.1 cc were associated with increased ≥ grade 2 GU toxicities (adjusted odds ratio [OR]: 1.14, *p* < 0.0001, and adjusted OR: 1.12, *p* < 0.0001, respectively) [77]. Similarly, according to a meta-analysis of 33 prospective studies applying radiotherapy with a focal boost (including both EBRT and brachytherapy), the urethra dose max was associated with ≥ grade 2 late GU toxicities (OR: 1.02, *p* < 0.001) [78]. Additionally, in an international survey of urethra-sparing prostate radiotherapy [79], 91% of experts recommend urethra-sparing with dose hotspot limitation in the setting of SBRT with a focal boost, while most of them did not employ specific dose constraints for the urethra when applying a conventional (58%) or moderate (49%) hypofractionation regimen. Although no clear consensus was reached regarding dose constraints to OARs due to high-level heterogeneity in EBRT schedules, 53% of the experts implemented a max dose (Dmax) of less than 90 Gy in EQD2 as a dose constraint for the urethra.

Regarding GI toxicities, in the post-hoc analysis of the FLAME phase 3 trial, D2 cc and D50% of the anorectum were related to increased ≥ grade 2 GI toxicities (adjusted OR: 1.17, p < 0.0001, and adjusted OR: 1.20, *p* < 0.0001, respectively) [80]. In addition, according to the aforementioned meta-analysis, the rectal dose max was associated with ≥ grade 2 acute GI toxicities (OR: 1.05, *p* < 0.001) [78].

## Treatment outcomes: oncological outcomes

Regarding conventional or moderately hypofractionated FB-SIB-IMRT, four prospective studies reported data with long-term follow-up, in which the 5-year biochemical relapse-free survival (bRFS) ranged from 92 to 98.5%. Among them, two reported comparative data with non-FB-SIB-IMRT. The phase 3 FLAME trial demonstrated 7% improvement in biochemical disease-free survival (5-year bRFS: 92 vs. 85%, respectively, HR: 0.45, 95% CI 0.28–0.71, *p* < 0.001) [6]. In addition, a difference was also observed both in local failure (adjusted HR: 0.33, 95% CI 0.14–0.80, *p* = 0.01) and regional plus distant metastatic failure (adjusted HR: 0.56, 95% CI 0.34–0.91, *p* = 0.02) [81]. Schild et al. also reported 24% improvement in the 10-year rate of biochemical control in patients who received FB-SIB-IMRT (82 Gy for IPDLs and 75.2 Gy for the entire prostate in 42 fractions) compared with a historical control receiving IMRT without a focal boost (*p* = 0.02)[82].

Regarding ultra-hypofractionated FB-SIB-IMRT, two prospective studies reported clinical data with a follow-up longer than 5 years, in which the 5-year bRFS rates were 93% [61] and 69% [37], respectively. Only one study reported comparative data of SBRT with vs. without a focal boost. Ong et al. compared clinical outcomes in 60 patients with low- or intermediate-risk PCa enrolled in two phase 2 studies, in which patients received 26 Gy in 2 fractions with or without focal dose-escalation up to 32 Gy [83]. There was no significant difference in BF (*p* = 0.1). Specifically, the 4-year cumulative incidence of BF was 8.3% (with focal boost) and 0% (without focal boost), respectively.

We previously performed a prospective pilot study of FB-SIB-IMRT using dose fractionation between moderate and ultra-hypofractionation (54 Gy for the prostate and 57 Gy for IPDLs in 15 fractions) [44]. The 5-year BF-free and clinical failure-free survival rates were 75.2 and 80.5%, respectively, with no patients developing local intraprostatic recurrence.

## Treatment outcomes: toxicity outcomes

Regarding conventional or moderately hypofractionated FB-SIB-IMRT, ≥ grade 2 GU and GI acute toxicities ranged from 20 to 56 and 0 to 14.8%, and ≥ grade 3 GU and GI acute toxicities ranged from 0 to 2.5 and 0 to 5%, respectively [6, 13–15, 26–28, 45, 59, 73, 74, 82, 84]. Only one study reported the occurrence of grade 4 acute GU toxicity (0.6%, urinary catheter) [27]; no grade 4 acute GI toxicities were reported. Four prospective studies reported late-toxicity data with a nearly 5-year follow-up, in which ≥ grade 2 GU and GI late toxicities ranged from 9.1 to 44 and 12.8 to 23.4%, and ≥ grade 3 GU and GI late toxicities ranged from 2 to 5.6 and 0 to 2.7%, respectively [6, 28, 59, 82]. Two studies reported the occurrence of grade 4 late GU toxicities (2.3 and 1.4%, respectively) [59, 82]; however, no grade 4 late GI toxicities were reported. Regarding ultra-hypofractionated FB-SIB-IMRT, ≥ grade 2 GU and GI acute toxicities ranged from 12.5 to 56.7 and 0 to 12.5%, ≥ grade 3 GU and GI acute toxicities ranged from 0 to 5 and 0%, and no grade 4 or higher GI toxicities were reported [29–39, 61]. Also, no grade 4 acute GU or GI toxicities were identified. There were three prospective studies which reported late-toxicity data with a nearly 5-year follow-up, in which ≥ grade 2 GU and GI late toxicities ranged from 12.1 to 28 and 3 to 14%, and ≥ grade 3 GU and GI late toxicities ranged from 0 to 2 and 0 to 1%, respectively [36, 37, 61]. No grade 4 late GU and GI toxicities were reported. When comparing toxicities between SBRT with vs. without a focal boost, although there was no significant difference in acute and late cumulative worst GU, GI, and sexual toxicities, regarding individual toxicities, there were higher rates of ≥ grade 1 acute and late urinary urgency and ≥ grade 1 acute constipation among those who received SBRT with a focal boost [83].

According to the aforementioned meta-analysis of 33 prospective studies regarding radiotherapy with a focal boost (including both EBRT and brachytherapy) [78], rates of acute ≥ grade 2 GU and GI toxicities were 25.3% (95% CI 19.1–32.8%) and 5.6% (95% CI 3.5–8.7%), and the pooled cumulative incidences of late ≥ grade 2 GU and GI toxicities were 21.1% (95% CI 16.7–26.3%) and 6.9% (95% CI 4.6–10.1%), respectively.

In our prospective study using dose fractionation between moderate and ultra-hypofractionation, ≥ grade 2 GU and GI acute toxicities were 26.9 and 7.7%, respectively, and no ≥ grade 3 toxicities were observed [44]. The 5-year cumulative incidence rates of grade 2 late GU and GI toxicities were 15.4 and 3.8%, respectively. No grade 3 or higher late GU or GI toxicities were noted.

## Future perspectives on FB-SIB-IMRT

Although several prospective datasets, including those from a phase 3 study, support the benefits of focal dose-escalation, optimal candidates for FB-SIB-IMRT have yet to be clearly established. For patients with low- or intermediate-risk PCa, currently available IMRT in combination with modern IGRT facilitated excellent disease control even without focal boosting. According to a phase 2 study of moderately hypofractionated IMRT (70 Gy in 28 fractions) in combination with prostate-position-based IGRT, the BF-free survival rate was 97.4% at 5 years among patients with intermediate-risk PCa [69]. For patients with high-risk PCa, there may remain room for improvement of tumor control via focal boosting [6, 85]. The population included in the aforementioned FLAME phase 3 trial primarily consisted of patients with high-risk PCa (84%) [6]. In its additional analysis, patients with high-risk characteristics, especially grade group 4–5 PCa, showed a low disease-free survival rate, while patients in grade group 1 showed a high disease-free survival rate even without focal boosting [85]. Additionally, when simulating D98% of 95 Gy was achieved with focal boosting, all risk groups showed a high disease-free survival rate. Conversely, the patients with much higher risk PCa, such as those with multiple unfavorable risks, are also more likely to show regional or distant metastasis, and so the benefit of intensifying local treatment may be modest due to undetectable metastasis. Therefore, accurate staging using novel imaging modalities, such as PSMA-PET/CT [12, 86] or whole-body diffusion-weighted MRI [87], may be particularly important for this risk group. To determine optimal candidates for FB-SIB-IMRT, further investigations, including the application of PSMA-PET/CT and development of risk models, are needed.

Prophylactic whole-pelvic radiation therapy (WPRT) might offer another solution to improve oncological outcomes, especially among patients with highly advanced PCa [88–90]. The POP-RT phase 3 trial, which compared whole-pelvic and prostate-only irradiation, demonstrated significant improvement of BF-free survival among patients with high- and very high-risk PCa (95.0 vs. 81.2% at 5 years, respectively, *p* < 0.0001), in which PSMA-PET/CT was used in approximately 80% for initial staging [89]. In addition, given the observation that the benefit of prophylactic WPRT is expected when high-dose irradiation is applied to the prostate, such as its combination with a high-dose-rate brachytherapy (HDR-BT) boost to the prostate [88, 91], a combination of FB-SIB-IMRT and prophylactic WPRT may be a promising method to improve oncological outcomes among patients with an unfavorable prognosis. Currently, this method is being tested in several early-phase prospective studies [28, 92–95]. To confirm the safety of this approach, the accumulation of clinical data with longer follow-up periods is needed.

## Conclusion

Based on current technological advances enabling this EBRT method, especially IGRT method, and accumulating evidence supporting the benefit of focal dose-escalation, FB-SIB-IMRT may become a standard IMRT method for PCa in the future. The expanding clinical application of this IMRT method, as well as further advance in IGRT, including oART, and combination of novel diagnostic modalities with higher-level accuracy, will hopefully improve oncological outcomes for patients with PCa.
